# Association of Time of Day With Functional Outcome in Intracerebral Hemorrhage

**DOI:** 10.1111/ene.70762

**Published:** 2026-09-19

**Authors:** Franziska Lieschke, Eng H. Lo, Emiri T. Mandeville, Christian Foerch, Elizabeth B. Klerman, Jeffrey L. Saver, Erik Maronde, Jan Hendrik Schaefer, Ferdinand O. Bohmann, Thorsten Steiner, Marco Stein, Björn Misselwitz, Daniel T. Marggrander

**Affiliations:** ^1^ Department of Neurology Goethe University Frankfurt, University Hospital Frankfurt/Main Germany; ^2^ Department of Neurology With Experimental Neurology Charité Universitätsmedizin Berlin Berlin Germany; ^3^ Department of Radiology, Neuroprotection Research Laboratories Harvard Medical School, Massachusetts General Hospital Boston Massachusetts USA; ^4^ Department of Neurology RKH Klinikum Ludwigsburg Ludwigsburg Germany; ^5^ Department of Neurology Harvard Medical School, Massachusetts General Hospital Boston Massachusetts USA; ^6^ David Geffen School of Medicine at UCLA, UCLA Comprehensive Stroke and Vascular Neurology Program University of California, Los Angeles Los Angeles California USA; ^7^ Dr. Senckenberg Institute for Anatomy II, Experimental Neurobiology Goethe University Frankfurt Frankfurt/Main Germany; ^8^ Department of Neurology Varisano Hospital Frankfurt Höchst Frankfurt/Main Germany; ^9^ Department of Neurology Heidelberg University Hospital Heidelberg Germany; ^10^ Department of Neurosurgery Justus Liebig University Giessen, University Hospital Giessen Giessen Germany; ^11^ Federal State Consortium of Quality Assurance Hesse Eschborn Germany; ^12^ Department of Congenital Heart Disease ‐ Pediatric Cardiology Deutsches Herzzentrum der Charité Berlin Germany; ^13^ Corporate Member of Freie Universität Berlin and Humboldt‐Universität Zu Berlin Charité Universitaetsmedizin Berlin Berlin Germany; ^14^ Department of Anesthesiology, Intensive Care and Pain Therapy Sana Hospital Offenbach Offenbach/Main Germany

**Keywords:** brain hemorrhage, chronobiology, circadian rhythm, ICH epidemiology, registries

## Abstract

**Background:**

The timing of ischemic stroke and intracerebral hemorrhage (ICH) onsets follow distinct daily patterns, but it remains unclear whether these patterns influence ICH outcomes.

**Methods:**

Consecutive data were obtained from the prospective stroke inpatient quality assurance registry of Hesse, Germany from 2015 to 2023. Patients with ICH were grouped according to the time of symptom onset: morning (5:00 AM to 10:59 AM), midday (11:00 AM to 4:59 PM), evening (5:00 PM to 10:59 PM), and night (11:00 PM to 4:59 AM). The primary outcome was global disability at discharge, analyzed using ordinal logistic regression. Secondary outcomes included mortality and complications during hospitalization. To account for potential confounding, the analysis incorporated both inverse probability weighting (IPW) and propensity score matching (PSM) based on baseline characteristics.

**Results:**

After exclusions, 5665 patients underwent final analysis. Peak ICH incidences occurred around 8:30 AM and 5:00 PM with a minimum at approximately midnight. Patients experiencing ICH during the evening and particularly at night had higher discharge disability, compared to those with symptom onset in the morning or midday. Unadjusted analyses found daily variations in mortality and in‐hospital complications that were no longer significant after adjustment by PSM or IPW.

**Conclusion:**

Our study confirms the daily pattern previously observed in ICH onset, with peak onset during daytime. Functional outcomes were worse in evening and night onset ICH patients. These findings underscore the potential for chronobiologically informed prevention and treatment strategies and the need for further research into time‐dependent pathophysiology and care delivery.

## Graphical abstract

In this registry study of 5665 patients with intracerebral hemorrhage (ICH), symptom onset times followed a distinct daily pattern with peaks in the morning and early evening. Patients with evening or nighttime ICH onset had worse functional outcomes at hospital discharge than those with morning or midday onset. Although unadjusted analyses suggested differences in mortality and in‐hospital complications, these associations were no longer significant after adjustment for baseline characteristics.

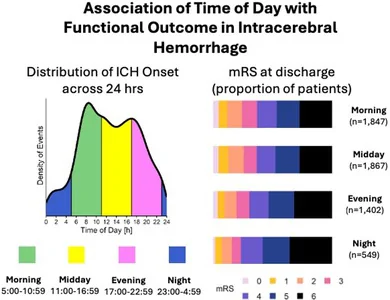

## 1 Introduction

The daily occurrence patterns of ischemic strokes, characterized most often by a single morning peak, have been extensively studied [1, 2]. For intracerebral hemorrhage (ICH), a similar morning peak but also a second evening peak has most often been described, suggesting influence by multiple time‐regulated (e.g., endogenous circadian and/or related to daily patterns of sleep/wake, rest/activity, feeding/fasting, time of medication or other behaviors) pathomechanisms [3, 4, 5, 6, 7, 8]. Previous studies have shown that the time of stroke onset plays an important role in presenting severity, disease course, and functional outcomes in acute ischemic strokes [9, 10]. Data on such circadian/daily influences on ICH are still insufficient.

Previous studies have highlighted potential circadian/daily variations in ICH incidence, clinical severity, and functional outcomes. For instance, data from the Takashima Stroke Registry indicated that ICHs occurring in the early morning hours were associated with lower symptom severity, as measured by the National Institutes of Health Stroke Scale (NIHSS); however, these cases also exhibited higher 30‐day mortality rates [11]. In a single‐center U.S. study involving 111 consecutive ICH patients, hematoma expansion was more frequently observed among those admitted in the morning (6:00 AM to 12:00 PM) [12]. Conversely, a recent multicenter U.S. study reported that patients admitted during nighttime hours tended to present with larger hematomas, which were associated with poorer prognoses [13].

The aim of this study is to characterize patients with ICH based on the time of ICH occurrence using consecutive, quasi population‐based, real‐life data from our mandatory state‐wide quality assurance registry. To this end, we compared ICH occurring at different times across the 24‐h day, specifically analyzing potential epidemiological differences, clinical characteristics, disease course, complications, and short‐term functional outcomes.

## 2 Methods

### 2.1 Patient Population

We obtained data from 119 hospitals in the federal state of Hesse, Germany participating in the required prospective stroke inpatient quality assurance registry LAGQH (www.lagqh.de). This registry encompasses the entire hospital network within Hesse, which serves a population of approximately 6.3 million residents [14]. Since data collection is anonymized and conducted as part of quality assurance measures, individual patient consent and ethical approval were not required. This study was conducted and reported in accordance with the STROBE (Strengthening the Reporting of Observational Studies in Epidemiology) and RECORD (REporting of studies Conducted using Observational Routinely‐collected Data) guidelines. Furthermore, all procedures performed in this study adhered to the ethical standards outlined in the Declaration of Helsinki.

### 2.2 Inclusion Criteria

Adults aged 18 years and older who were admitted between 2015 and 2023 with the encoded diagnosis I61 (atraumatic intracerebral hemorrhage) based on the International Classification of Diseases, 10th Version (ICD‐10) were included. Additional inclusion criteria required a documented symptom onset (last known well time) and complete data on medical history, clinical course, and functional status at presentation and discharge or transfer (Figure S1).

### 2.3 Clinical Parameters

Data extracted from the registry included baseline characteristics, the time of admission, time between symptom onset (last known well time) and admission, the calculated time of onset, physical examination on admission, medical history risk factors and prior functional status, hemorrhage location, initiated therapies, complications sustained including mortality, and functional status at discharge, among other variables.

### 2.4 Patient Grouping and Additional Exclusion Criteria

We distinguished patients with symptom onset during the morning (5:00 AM to 10:59 AM), midday (11:00 AM to 4:59 PM), evening (5:00 PM to 10:59 PM), and night (11:00 PM to 4:59 AM), as calculated from the time of admission and reported delay from symptom onset. As recollected time frames from symptom onset are known to be reported more inaccurately the longer admission is delayed [15, 16], we eliminated patients admitted more than nine hours since symptom onset. We analyzed time‐of‐onset data both by these time‐of‐day categories and in a continuous manner (graphically depicted as kernel density estimates).

### 2.5 Statistical Adjustment for Confounding

To control for confounding, inverse probability weighting (IPW) was applied based on à priori available baseline characteristics including age, sex, time from onset to hospital admission, previous medical comorbidities and the presence of advance healthcare directives. The distribution of the inverse probability weights is shown in Figures S2 and S3, while Figure S4 displays the covariate balance diagnostics. A secondary adjustment approach employed propensity score matching (PSM) to construct a balanced cohort. PSM was performed using the same baseline variables as for the IPW (i.e., age, sex, time to hospital admission, previous medical comorbidities and the presence of advance healthcare directive). Patients were grouped according to a binary time split (daytime from 7:00 AM to 10:59 PM vs. nighttime from 11:00 PM to 6:59 AM). This 16‐ vs. 8‐h diurnal vs. nocturnal split was consistent with the Leducq Consortium International pour la Recherche Circadienne sur l'AVC Effects in Stroke (CIRCA) consensus recommendations [17], based on the observation that most healthy people typically have an 8‐h rest period with sleep onset at 11 pm [18, 19, 20]. Because we anticipated more patients in the daytime group (16 h) compared to the nighttime group (8 h), we matched patients using a 2:1 ratio. IPW was used for modeling the four groups as defined above from a multinomial model to incorporate all patients in the dataset. Propensity score matching was chosen for the binomial split as a more traditional procedure to control for confounders between two groups and to verify results from the IPW analysis while avoiding overfitting or extreme weights, with the disadvantage of discarding unmatched patients.

### 2.6 Outcome Measures

The primary outcome was global disability status at discharge, assessed using the modified Rankin Scale (mRS). Differences in mRS scores across time‐of‐onset groups were examined both before and after adjustment via IPW and PSM. Additionally, ordinal logistic regression was employed [21]. Secondary outcomes included in‐hospital mortality and complications during hospitalization.

### 2.7 Statistical Analysis

Statistical analysis was performed using GNU R 4.4.1 and GraphPad PRISM 10.3.1. Normally distributed data are depicted as mean ± standard deviation (SD), continuous data without normal distribution as median and interquartile range (Q1‐Q3), and frequencies of categorical data as percentage. Continuous data were compared using student's *t*‐test or the Wilcoxon‐Mann–Whitney‐test or Kruskal‐Wallis‐tests, depending on normality, which we assessed with the Kolmogorov–Smirnov‐test. Categorical variables were compared using the *χ* ^2^‐test. Ordered logistic regression of the primary outcome was modeled with inverse probability weights. The fit of the regression models was evaluated using Nagelkerke's Pseudo‐R^2^. We considered *p*‐values < 0.05 to be statistically significant.

## 3 Results

### 3.1 Daily Pattern of ICH Onset

**FIGURE 1:**
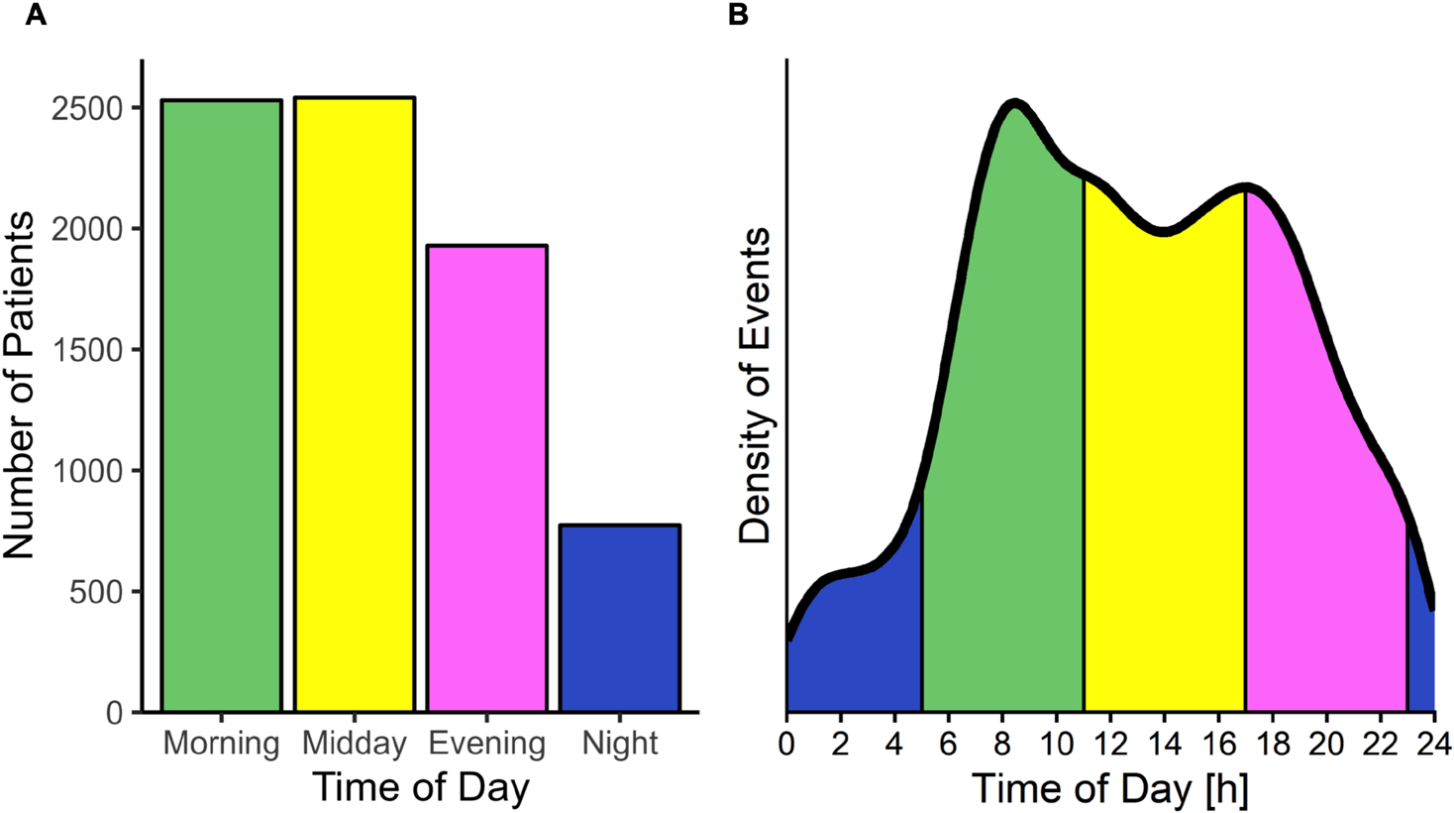
Distribution of ICH‐onset (estimated using the time of admission and reported time delay since symptom onset) according to the time of day. A: Histogram. B: Density diagram. Estimated ICH‐onset time groupings were Morning: 5:00 AM to 10:59 AM, Midday: 11:00 AM to 4:59 PM, Evening: 5:00 PM to 10:59 PM, Night: 11:00 PM to 4:59 AM. ICH, intracerebral hemorrhage.

A total of 12,187 patients with ICH were reported to the database between 2015 and 2023. In 7770 of these patients, the time of symptom onset (or, if unwitnessed the last known well time) could be calculated with sufficient precision (known symptom onset time no longer than 9 h from hospital admission) to allocate them according to the time of day categories: in 2529 of the reported patients ICH onset was during the morning (5:00 AM to 10:59 AM), in 2540 patients, ICH occurred during the midday (11:00 AM to 4:59 PM), in 1928 during the evening (5:00 PM to 10:59 PM), and in 773 during the night (11:00 PM to 4:59 AM) (Figure 1A). The continuous variation in ICH onset time of day is shown in Figure 1B. Peak incidence occurred at ~8:30 AM with a later maximum at ~5:00 PM; minimum incidence occurred at approximately midnight. Only 10% of ICH were during the night (11:00 PM to 4:59 AM).

### 3.2 Patient Characteristics According to Time‐Of‐Day Categories

Among the 7770 patients with a known symptom onset, data on medical history, disease course, presenting functional status as well as functional status at discharge or transfer were available for 5665 patients. The baseline characteristics, physical examination on admission, initiated therapies, and previous risk factors of patients in the four times of day categories are shown in Table 1.

**TABLE 1:** Baseline characteristics, initiated therapies, and medical history according to the time‐of‐day onset groups.

	Morning (*n* = 1847)	Midday (*n* = 1867)	Evening (*n* = 1402)	Night (*n* = 549)	*p*	IPW‐adjusted *p*
Characteristics at admission						
Median age [years]	77 (66–83)	75 (63–82)	76 (64–83)	75 (62–82)	**< 0.01**	
Median time to admission [hours]	2 (2–4)	2 (1–3)	2 (1–3)	2 (2–4)	**< 0.01**	
Sex (male/female) [%]	988/859 (53.5/46.5)	1020/847 (54.6/45.4)	760/642 (54.2/45.8)	312/237 (56.8/43.2)	0.58	
Median GCS at admission	13 (9–15)	13 (8–15)	12 (7–15)	12 (5–15)	**< 0.01**	**< 0.01**
Advance healthcare directive present (%)	513 (27.8)	509 (27.3)	448 (32.0)	187 (34.1)	**< 0.01**	
Paresis of extremities at admission (%)	1328 (71.9)	1268 (67.9)	970 (69.2)	368 (67.0)	**0.03**	0.05
Aphasia at admission (%)	613 (33.2)	678 (36.3)	477 (34.0)	163 (29.7)	**0.02**	**< 0.01**
Dysarthria at admission (%)	852 (46.1)	834 (44.7)	633 (45.2)	237 (43.2)	0.63	0.68
Dysphagia at admission (%)	646 (35.0)	627 (33.6)	507 (36.2)	194 (35.3)	0.48	**< 0.01**
Therapeutic decisions and clinical course						
Surgical therapy (%)	308 (16.7)	337 (18.1)	201 (14.3)	96 (17.5)	**0.03**	0.32
Mechanical ventilation (%)	502 (27.2)	578 (31.0)	499 (35.6)	234 (42.6)	**< 0.0001**	**< 0.01**
Extraventricular drainage (%)	264 (14.3)	304 (16.3)	260 (18.6)	106 (19.3)	**0.003**	**0.01**
Admission to intensive care unit (%)	715 (38.7)	806 (43.2)	661 (47.2)	287 (52.3)	**< 0.0001**	**< 0.01**
Admission to stroke unit (%)	1324 (71.7)	1246 (66.7)	871 (62.1)	325 (59.2)	**< 0.0001**	**< 0.01**
Medical history						
History of hypertension (%)	1565 (84.7)	1477 (84.5)	1182 (84.3)	472 (86.0)	0.82	
History of atrial fibrillation (%)	420 (22.7)	367 (19.7)	304 (21.7)	128 (23.3)	0.09	
History of previous cerebrovascular event (%)	375 (20.3)	332 (17.8)	279 (19.9)	126 (23.0)	**0.04**	
History of diabetes mellitus (%)	371 (20.1)	314 (16.8)	243 (17.3)	110 (20.0)	**0.03**	

*Note:* The groups are defined by the estimated ICH‐onset time (morning: 5:00 AM to 10:59 AM, midday: 11:00 AM to 4:59 PM, evening: 5:00 PM to 10:59 PM, night: 11:00 PM to 4:59 AM). The *p*‐values < 0.05 that are considered statistically significant are depicted in bold font.

Abbreviations: GCS, Glasgow Coma Scale; IPW, Inverse Probability Weighting.

Notable differences were identified across the patient groups. Patients experiencing symptom onset in the morning were generally older. The interval from symptom onset to hospital admission was shortest among patients whose symptoms began at midday or in the evening. Advance healthcare directives were more commonly documented in evening and nighttime onset groups. In terms of clinical presentation, Glasgow Coma Scale (GCS) scores suggested greater severity in patients with evening and nighttime symptom onset. Paresis was most frequently observed in the morning‐onset group, while aphasia was most prevalent among those with midday onset. Admissions to the intensive care unit, mechanical ventilation, and utilization of external ventricular drainage were more common in patients with evening, and particularly nighttime, onset. Furthermore, preexisting conditions such as diabetes mellitus and previous cerebrovascular events were more frequently noted in patients with symptoms arising during the night and morning hours. ICH locations did not differ significantly (*p* = 0.84) between the four groups by time of day (Figure S5).

### 3.3 Functional Outcomes

**FIGURE 2:**
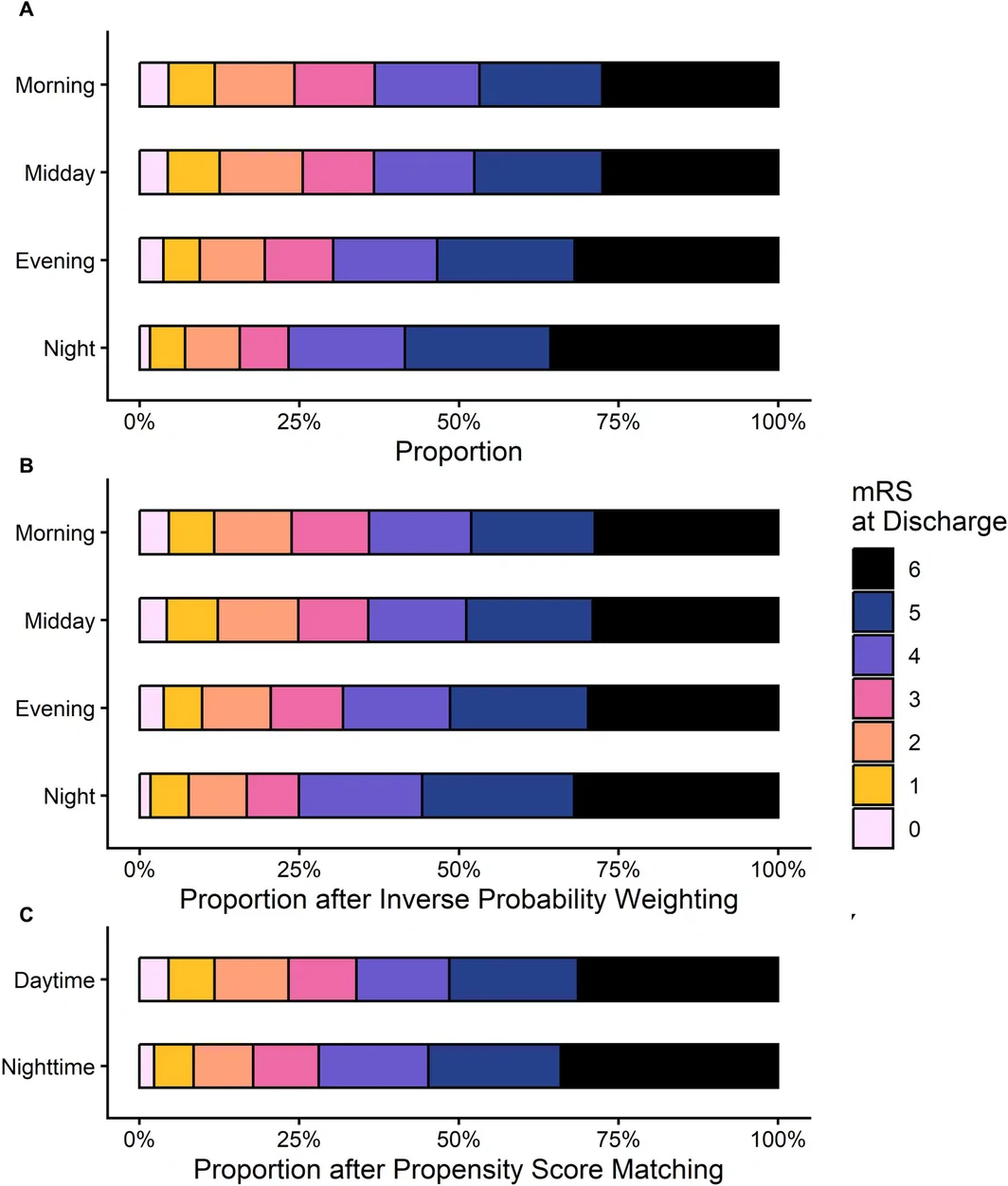
Frequency distribution of the global disability outcome based on modified Rankin scale (mRS) scores at discharge (displayed as %) in patients with symptom onset during either the morning (5:00 AM to 10:59 AM), midday (11:00 AM to 4:59 PM), evening (5:00 PM to 10:59 PM) or the night (11:00 PM to 4:59 AM) in the unadjusted cohort (A, *p* < 0.01) and after IPW‐adjustment (B, *p* < 0.01). C: Frequency distribution of the mRS at discharge in patients with symptom onset during either the day (7:00 AM–10:59 PM) or night (11:00 PM–6:59 AM) after propensity score matching (PSM) adjusted for age, sex, time to hospital admission, pre‐existing medical comorbidities, and the presence of advance healthcare directives (C, *p* < 0.01).

Patients with ICH onset during the nighttime showed poorer functional outcomes at discharge compared to patients with ICH onset during the daytime: median mRS at discharge was 5 (3–6) in the evening onset group, 5 (4–6) in the night onset group, 4 (3–6) in the morning onset group and 4 (2–6) in the midday onset group (*p* < 0.0001 in the unadjusted analysis, Figure 2A; *p* < 0.001 after IPW‐adjustment, Figure 2B; Table 2). Functional outcome at discharge was significantly poorer in patients who developed secondary intraventricular hemorrhage (sIVH: median 6, IQR 5–6) compared to patients without sIVH (median 4, IQR 3–6, *p* < 0.001).

**TABLE 2:** Functional outcomes according to the time‐of‐day onset groups.

	Morning (*n* = 1847)	Midday (*n* = 1867)	Evening (*n* = 1402)	Night (*n* = 549)	*p*	IPW‐adjusted *p*
mRS at discharge						
median mRS	4 (3–6)	4 (2–6)	5 (3–6)	5 (4–6)	**< 0.0001**	**< 0.01**
mean (±SD)	3.97 (±1.8)	3.96 (±1.8)	4.22 (±1.8)	4.46 (±1.6)		
mRS (%)					**< 0.0001**	**< 0.01**
mRS 0	84 (4.6)	82 (4.4)	52 (3.7)	9 (1.6)		
mRS 1	133 (7.2)	152 (8.1)	80 (5.7)	30 (5.5)		
mRS 2	231 (12.5)	243 (13.0)	143 (10.2)	47 (8.6)		
mRS 3	232 (12.6)	208 (11.1)	150 (10.7)	42 (7.7)		
mRS 4	303 (16.4)	293 (15.7)	228 (16.3)	100 (18.2)		
mRS 5	355 (19.2)	375 (20.1)	302 (21.5)	125 (22.8)		
mRS 6	509 (27.6)	514 (27.3)	447 (31.9)	196 (35.7)		

*Note:* The groups are defined by the estimated ICH‐onset time (morning: 5:00 AM to 10:59 AM, midday: 11:00 AM to 4:59 PM, evening: 5:00 PM to 10:59 PM, night: 11:00 PM to 4:59 AM). The *p*‐values < 0.05 that are considered statistically significant are depicted in a bold font.

Abbreviations: IPW, Inverse Probability Weighting; mRS, modified Rankin Scale; SD, standard deviation.

To validate these findings, we conducted PSM adjusted for age, sex, time to hospital admission, pre‐existing medical comorbidities, and the presence of advance healthcare directives, and incorporating a binary time‐of‐onset classification (daytime: 7:00 AM–10:59 PM; nighttime: 11:00 PM–6:59 AM). PSM yielded a subset of 3096 patients, with 1032 experiencing ICH onset at night and 2064 during the daytime, which were well‐balanced across baseline characteristics (Table S1). In the matched cohorts, the median discharge mRS score was similar between patients with nighttime and daytime ICH onset (5 (3–6) vs. 5 (3–6), respectively); however the overall distribution of outcomes remained significantly different (*p* < 0.01; Figure 2C).

Ordinal logistic regression (Table 3) confirmed nighttime onset as a risk factor contributing to higher mRS scores (OR 1.3). Other factors significantly contributing to increased mRS scores at discharge included higher age (OR 1.02), presence of an advance healthcare directive (OR 64.4), previous cerebrovascular events (OR 1.2), the need for mechanical ventilation (OR 3.0) or intensive care (OR 1.5), and presence of paresis (OR 2.6), dysarthria (OR 1.2) or dysphagia (OR 1.9) at admission. Factors that were associated with decreased mRS scores were longer admission times (OR 0.9), higher GCS scores (OR 0.8), and use of surgical therapies (OR 0.5).

**TABLE 3:** Ordered logistic regression of modified Ranking scale (mRS) at discharge adjusted by inverse probability weighting, *R* ^2^ = 0.679.

	Odds ratio (95% confidence interval)	*p*
Predictor		
Event during the morning	1.05 (0.92–1.19)	0.5
Event during the evening	1.05 (0.92–1.21)	0.47
Event during the night	1.26 (1.04–1.53)	**0.02**
Time to hospital admission (hours)	0.94 (0.91–0.98)	**0.001**
Age (years)	1.02 (1.01–1.02)	**< 0.001**
Female sex	1.05 (0.94–1.17)	0.39
GCS at admission	0.80 (0.78–0.81)	**< 0.001**
Advance healthcare directive present	64.60 (52.67–79.56)	**< 0.001**
History of atrial fibrillation	1.14 (1.00–1.30)	0.05
History of arterial hypertension	0.96 (0.82–1.13)	0.63
History of cerebrovascular insult	1.15 (1.01–1.32)	**0.04**
History of diabetes	1.12 (0.98–1.28)	0.1
Extraventricular drainage	0.96 (0.81–1.15)	0.69
Surgical therapy	0.52 (0.42–0.63)	**< 0.001**
Endovascular therapy	1.27 (0.84–1.93)	0.25
ICU admission	1.47 (1.25–1.73)	**< 0.001**
Mechanical ventilation	3.03 (2.47–3.72)	**< 0.001**
Stroke unit admission	0.73 (0.84–1.93)	0.25
Paresis of extremities at admission	2.64 (2.32–3.00)	**< 0.001**
Aphasia at admission	1.09 (0.97–1.22)	0.15
Dysarthria at admission	1.32 (1.17–1.48)	**< 0.001**
Dysphagia at admission	1.93 (1.70–2.19)	**< 0.001**
Intercept		
mRS 1	0.02 (0.01–0.03)	**< 0.001**
mRS 2	0.07 (0.05–0.12)	**< 0.001**
mRS 3	0.24 (0.15–0.38)	**< 0.001**
mRS 4	0.59 (0.37–0.93)	**0.02**
mRS 5	2.26 (1.43–3.58)	**< 0.001**
mRS 6	24.19 (15.23–38.41)	**< 0.001**

*Note:* The *p*‐values < 0.05 that are considered statistically significant are depicted in a bold font.

Abbreviations: GCS, Glasgow Coma Scale; ICU, Intensive Care Unit.

### 3.4 Secondary Outcomes (Including Mortality and Complications Sustained During Hospital Stay)

Patients with evening, and especially nighttime, symptom onset exhibited higher rates of mortality, cerebral edema, and pneumonia; however, after inverse probability weighting (IPW) adjustment, only the association with pneumonia remained statistically significant (Table 4). Although patients with ICH onset during the day developed sIVH slightly more often than those with a nightly onset, this difference did not reach statistical significance (Table 4). Mortality was significantly higher among patients who developed sIVH than among those without sIVH (65.3% vs. 29.0%, *p* < 0.001).

**TABLE 4:** Complications sustained during hospital stay according to the time‐of‐day onset groups.

	Morning (*n* = 1847)	Midday (*n* = 1867)	Evening (*n* = 1402)	Night (*n* = 549)	*p*	IPW‐adjusted *p*
Complications						
Mortality (%)	514 (27.8)	517 (27.7)	450 (31.2)	199 (36.3)	**< 0.01**	0.43
Cerebral edema (%)	126 (6.8)	127 (6.8)	114 (8.1)	56 (10.2)	**0.03**	0.36
Secondary intraventricular hemorrhage (%)	30 (1.6)	39 (2.1)	23 (1.6)	6 (1.1)	0.41	0.21
Bleeding progression or rebleeding including secondary intraventricular hemorrhage (%)	70 (3.8)	93 (5.0)	56 (4.0)	19 (3.5)	0.21	0.33
Pneumonia (%)	308 (16.7)	337 (18.1)	273 (19.5)	119 (21.7)	**0.03**	**0.02**
Epileptic seizures (%)	58 (3.1)	66 (3.5)	32 (1.3)	13 (2.4)	0.16	0.10
Hydrocephalus (%)	66 (3.6)	79 (4.2)	65 (4.6)	20 (3.6)	0.44	0.64

*Note:* The groups are defined by the estimated ICH‐onset time (morning: 5:00 AM to 10:59 AM, midday: 11:00 AM to 4:59 PM., evening: 5:00 PM to 10:59 PM, night: 11:00 PM to 4:59 AM). The *p*‐values < 0.05 that are considered statistically significant are depicted in a bold font.

Abbreviation: IPW, Inverse Probability Weighting.

When comparing the secondary outcomes after PSM, none of them remained statistically significant different between nighttime and daytime onset groups. The outcomes of the matched patients are depicted in Table S2.

## 4 Discussion

This study demonstrates a pronounced daily variation in ICH onset, with a primary peak in the morning and a secondary peak in the afternoon, aligning with previously reported patterns [3, 6, 7, 13]. Functional outcomes, as measured by the modified Rankin Scale (mRS), were poorer in patients with symptom onset during the evening and night; however, mortality and rates of in‐hospital complications except for pneumonia did not differ significantly across time‐of‐day groups following adjustment for confounders using IPW and PSM.

Unlike prior studies limited by moderate sample sizes and selective cohorts, our analysis includes a large, broadly representative patient population. Initially observed higher mortality among nighttime‐onset patients was likely driven by baseline imbalances, particularly the disproportionate presence of advance care directives in this group. Notably, the presence of such directives emerged as the strongest predictor of poor outcome. Advance care directives are decisive in clinical decision‐making as they may preclude life‐prolonging interventions once complications that limit neurological rehabilitation occur, thereby determining the subsequent course of treatment [22, 23, 24, 25, 26]. After accounting for this and other covariates through IPW and PSM, nighttime onset was no longer associated with increased mortality. Other factors such as older age and comorbidities also varied by time of onset and likely contributed to outcome disparities independent of diurnal timing. Consistent with our findings, a pooled analysis of the Intensive Blood Pressure Reduction in Acute Cerebral Hemorrhage Trials (INTERACT) involving 2904 patients [27] showed that ICH onset between late afternoon and early morning (3 PM to 8 AM) was associated with greater clinical severity, reflected by lower admission GCS scores; however, functional outcomes at 90 days (mRS) were not significantly influenced by the time of onset. Interestingly, longer delays from symptom onset to hospital admission were associated with better functional outcomes, likely reflecting delayed recognition in cases with milder initial syndrome severity, slower hemorrhage expansion, or atypical presentations [28, 29, 30]. However, since the initial syndrome severity is a key determinant of prognosis [31, 32, 33], it is not unexpected that patients with daytime onset of symptoms generally experience more favorable outcomes.

ICH is commonly associated with intraventricular hemorrhage (IVH), which is an independent predictor of mortality and poor functional outcome [34, 35, 36, 37]. Unfortunately, intraventricular extension of ICH already present on the initial imaging was not systematically recorded. In contrast, secondary IVH (sIVH: defined as delayed ventricular extension of the hematoma during the clinical course) was consistently recorded as a complication. Patients with sIVH demonstrated a significantly poorer functional outcome and higher mortality at discharge compared to patients without sIVH, consistent with previous studies [38, 39, 40]. No significant differences were detectable in the rate of sIVH across the four time‐of‐day onset groups. Furthermore, previous literature demonstrated that clinical outcomes after ICH differ substantially according to hemorrhage location [41, 42, 43, 44, 45, 46]. In our study, ICH locations did not differ significantly between the four time‐of‐day onset groups. Thus, potential chronobiological differences between distinct ICH locations or subtypes warrant dedicated investigation in future studies.

We also observed longer median delays to hospital admission during the night and early morning compared to midday and evening ICH symptom onset. This underscores the need to optimize stroke pathways and reduce delays in recognition, transport, and treatment initiation especially during nighttimes [47, 48]. Historical data from our registry (2003–2006), collected during the rollout of intravenous thrombolysis for ischemic stroke, suggest that nighttime or weekend care did not compromise treatment quality in general [49, 50]. However, surgical management of ICH may be more susceptible to variability in nighttime staffing, particularly regarding senior neurosurgeon availability [51, 52]. Although some evidence suggests this effect may be limited [53, 54, 55, 56], we could not assess time‐sensitive variables such as treatment delays or staff availability due to the retrospective nature of the study. These factors remain potential contributors to outcome variability and merit further investigation.

Hajdu et al. previously noted improved endovascular treatment outcomes among patients with acute ischemic stroke treated in the morning, without identifying baseline or hospital factors that might explain this observation [57]. Similarly, Ryu et al. found better functional outcomes with morning onset in a large cohort of patients with ischemic stroke and attributed the finding in part to slower ischemic stroke progression during the day, a finding supporting by multimodal imaging study [58, 59]. Findings like this and ours underscore the role of daily biological and behavioral rhythms in stroke pathophysiology. Temporal fluctuations in blood pressure, coagulation profiles, endothelial function and behavioral transitions (e.g., sleep‐to‐wake, fasting‐to‐eating, inactivity‐to‐activity) may contribute to increased vulnerability [60, 61]. While the potential time‐of‐day impact on hematoma expansion e.g., due to an increased blood–brain‐barrier vulnerability [62] was beyond the scope of the present study, this remains a plausible and clinically significant factor. Given its potential implications for patient prognosis, further investigation in this area is warranted. Elevated arterial blood pressure is a known independent predictor of poor outcome [63, 64], and early reduction may limit hematoma expansion [65, 66, 67]. Morning and afternoon surges in blood pressure could influence both ICH onset and hematoma growth, contributing to the observed daily variation. These insights support the potential utility of chronobiologically guided interventions, such as targeted nocturnal blood pressure control. The prognostic impact of blunted nocturnal blood pressure dipping in cardiovascular disease further supports this hypothesis [68, 69]. Future studies should aim to clarify the pathophysiological underpinnings of daily variation in ICH and evaluate time‐targeted strategies for prevention and management.

Despite the strengths of a large, prospectively maintained registry and robust statistical adjustment, our study has several limitations. First, the observational design precludes definitive causal inference. Although we implemented IPW and PSM to minimize bias, unmeasured confounders may still influence the findings. Important variables were missing helping to understand the severity of ICH such as the subtypes of ICH, ICH size and location and prior use of anticoagulants and antiplatelets, which may constitute important confounders and were not controlled or adjusted for. Moreover, symptom onset time was estimated based on reported delays and admission timestamps; this introduces potential recall bias and uncertainty especially for patient reported times [15]. To mitigate potential recall biases, therefore, only neurologist‐determined times for symptom onset were captured. To ensure data quality, patients with incomplete or imprecise timing information were excluded, potentially introducing selection bias. Especially patients with unknown symptom onset may differ systematically from those with known symptom onset and may even present with greater severity (e.g., unwitnessed events, decreased level of consciousness), which may potentially reflect distinct clinical pathways and outcomes. However, accurate classification of time‐of‐day exposure was not feasible without a reliable symptom onset estimate and the inclusion of such patients would therefore introduce substantial misclassification, likely biasing results in an unpredictable manner. The study was conducted using data from a regional registry in Hesse, Germany. Although the dataset is representative of the German healthcare system including 119 hospitals from rural community centers to tertiary academic institutions, our findings may not be generalizable to regions with different healthcare infrastructures or population demographics. Additionally, the registry lacked granularity in documenting specific treatment timelines (e.g., door‐to‐imaging or decision‐to‐intervention times), limiting our ability to assess operational contributors to outcome disparities. Lastly, outcome assessment was limited to discharge mRS and in‐hospital complications. These measures, while important, do not fully capture long‐term functional recovery or quality of life, and patients with similar discharge mRS scores may experience divergent trajectories post‐discharge. When interpreting mortality as an outcome, it is important to distinguish between neurological causes of death (e.g., elevated intracranial pressure or hydrocephalus) and non‐neurological causes, such as systemic complications (e.g., pneumonia) or other serious conditions (e.g., pulmonary embolism, cardiac events). Our dataset did not allow for such differentiation, as no assessment was performed to determine whether the recorded complications were directly related to mortality. This constitutes a further limitation constraining the clinical relevance of the findings.

## 5 Conclusion

This large‐scale analysis reveals a distinct time‐of‐day pattern in ICH incidence, with peak onset occurring during the morning and a second smaller peak in the early evening around 5:00 PM. Only 10% of ICH were during the night. Patients with evening and nighttime onset experienced worse functional outcomes, however in‐hospital complications and mortality were not independently associated with time of day after statistical adjustment for baseline differences. These findings highlight the need for future prospective and mechanistic studies to disentangle the effects of biological rhythms (i.e., circadian rhythms) from systemic care factors (e.g., time‐of day factors related to behaviors), and to develop evidence‐based strategies that ensure consistent stroke care quality around the clock. A better understanding of how circadian biology influences secondary brain injury mechanisms such as hematoma expansion, neuroinflammation, and secondary brain injury could help to identify times of increased vulnerability and responsiveness to therapies. In the future, this may support chronotherapeutic approaches in which interventions such as blood pressure control, hemostatic therapy, surgical evacuation, or anti‐inflammatory treatments are optimized according to biological timing, which could contribute to more personalized management strategies and potentially improve patient outcomes. In addition, incorporating symptom onset time into prognostic models may improve prediction of clinical course and functional outcome.

## Author Contributions

**Franziska Lieschke:** conceptualization, investigation, funding acquisition, writing – original draft, methodology, validation, visualization, writing – review and editing, formal analysis, project administration, data curation. **Eng H. Lo:** supervision, conceptualization, writing – review and editing, formal analysis. **Emiri T. Mandeville:** writing – review and editing. **Christian Foerch:** writing – review and editing. **Elizabeth B. Klerman:** writing – review and editing, formal analysis. **Jeffrey L. Saver:** supervision, writing – review and editing, formal analysis. **Erik Maronde:** writing – review and editing, supervision, funding acquisition. **Jan Hendrik Schaefer:** writing – review and editing. **Ferdinand O. Bohmann:** writing – review and editing. **Thorsten Steiner:** writing – review and editing. **Marco Stein:** writing – review and editing. **Björn Misselwitz:** resources, data curation, writing – review and editing. **Daniel T. Marggrander:** conceptualization, investigation, methodology, validation, visualization, writing – review and editing, formal analysis, data curation, supervision.

## Funding

The authors gratefully acknowledge funding support from the Faculty of Medicine, Goethe University Frankfurt (Junior Clinician Scientist scholarship to FL).

## Conflicts of Interest

F.L. reports speaker honoraria from Laerdal. C.F. reports speaker honoraria and honoraria for participating in advisory boards from Alexion, Bristol Myers Squibb, Novartis, Teva, Merck, Sanofi Genzyme, and Roche. F.O.B. reports speaker honoraria from Laerdal, AstraZeneca, Bristol‐Myers‐Squibb, Pfizer, Medtronic and grants and personal fees from Alexion, AstraZeneca, Stryker Neurovascular, Boehringer Ingelheim. E.B.K. (i) consulting: Buck Institute for Research on Aging; Circadian Therapeutics, National Sleep Foundation. (ii) honoraria, Sleep Research Society, (iii) travel support: Lorentz Center, Sante Fe Institute, Sleep Research Society, Society for Research in Biological Rhythms, World Sleep Foundation; (iv) other: Partner is founder and CSO of Chronsulting.

## Supporting information


**Figure S1:** Study flow chart.
**Figure S2:** Distribution of inverse probability weights among all 5665 patients analyzed (median 0.98, range 0.39–1.87).
**Figure S3:** Distribution of inverse probability weights across the four time‐of‐day groups.
**Figure S4:** Covariate balance diagnostics (A) for the IPTW analysis visualizing the maximum absolute standardized mean difference across treatment pairs before and after adjustment, and (B) Distribution of absolute standardized mean difference before and after PSM, both showing improved covariate balance.
**Figure S5:** Distribution of anatomical ICH locations across the four time‐of‐day onset groups (*p* = 0.84).
**Table S1:** Comparison of baseline characteristics, initiated therapies, and medical history between the daytime (i.e., 7:00 AM to 10:59 PM) and nighttime (i.e., 11:00 PM to 06:59 AM) onset groups after propensity score matching. Because we anticipated more patients in the daytime group (16 h) compared to the nighttime group (8 h), we matched patients using a 2:1 ratio. Continuous variables without a normal distribution are presented as median (interquartile range). Categorical variables are expressed as number (*n*) with percentage (%) in parentheses. The *p*‐values < 0.05 that are considered statistically significant are depicted in a bold font. Abbreviations: GCS = Glasgow Coma Scale. ^a^Wilcoxon‐Mann–Whitney‐test, ^b^ *χ* ^2^‐test.
**Table S2:** Outcomes according to the time of day of ICH onset after propensity score matching. The nighttime group summarizes patients suffering from ICH in the evening or night (11:00 PM‐ 6:59 AM); the daytime group summarizes patients with ICH onset during the morning or midday (7:00 AM‐ 10:59 PM).

## Data Availability

The data that support the findings of this study are available upon request from the corresponding author after permission by the data holders of the included regional quality assurance projects. The dataset was specifically pooled for this study with approval from each participating regional quality assurance project.
