# Supplementary material for: Association of Time of Day With Functional Outcome in Intracerebral Hemorrhage
Source: Eur J Neurol. 2026 Sep 19;33(9):e70762. doi: 10.1111/ene.70762 (PMC13589243; doi:10.1111/ene.70762)
Supplement: Supplementary file 1 [file ENE-33-e70762-s001.docx]

**Supplemental Material**

**Fig. S1:** Study flow chart.

**Fig. S2:** Distribution of inverse probability weights among all 5665 patients analyzed (median 0.98, range 0.39-1.87).

**Fig. S3:** Distribution of inverse probability weights across the four time-of-day groups.

**Fig. S4:** Covariate balance diagnostics (A) for the IPTW analysis visualizing the maximum absolute standardized mean difference across treatment pairs before and after adjustment, and (B) Distribution of absolute standardized mean difference before and after PSM, both showing improved covariate balance.

**Supplemental Analysis (ICH locations)**

The anatomical location of the intracerebral hemorrhage was available for a subset of 724 patients. Overall, hemorrhages were located in the basal ganglia in 275 patients (38.0%), lobar regions in 189 (26.1%), the thalamus in 72 (9.9%), the cerebellum in 56 (7.7%), multiple locations in 46 (6.4%), the brainstem in 45 (6.2%), and were classified as primary intraventricular hemorrhage in 41 patients (5.7%).

**Fig. S5: Distribution of anatomical ICH locations across the four time-of-day onset groups** (p= 0.84).

|  | **daytime onset (n= 2,064)** | **nighttime onset**  **(n=1,032)** | **p-value** |
| --- | --- | --- | --- |
| **Characteristics at admission** |  |  |  |
| Median age [years] | 75 (63-82) | 75 (63-82.25) | 0.75^a^ |
| Median time to admission [hours] | 3 (2-4) | 3 (2-4) | 0.92^a^ |
| Sex (male/female) (%) | 1185/879 (57.4/42.6) | 576/456 (55.8/44.2) | 0.42^b^ |
| Median GCS at admission | 12 (7-15) | 12 (6-15) | 0.50^a^ |
| Advance healthcare directive present (%) | 619 (30.0) | 314 (30.4) | 0.84^b^ |
| Paresis of extremities at admission (%) | 1440 (69.8) | 717 (69.5) | 0.90^b^ |
| Aphasia at admission (%) | 742 (35.9) | 325 (31.5) | **0.02^b^** |
| Dysarthria at admission (%) | 922 (44.7) | 461 (44.7) | 1^b^ |
| Dysphagia at admission (%) | 695 (33.7) | 354 (34.3) | 0.76^b^ |
| **Therapeutic decisions and clinical course** |  |  |  |
| surgical therapy (%) | 159 (7.7) | 93 (9.0) | 0.24^b^ |
| mechanical ventilation (%) | 636 (32.6) | 342 (35.8) | 0.10^b^ |
| extraventricular drainage (%) | 322 (15.6) | 156 (15.1) | 0.77^b^ |
| admission to intensive care unit (%) | 854 (43.8) | 445 (46.6) | 0.17^b^ |
| admission to stroke unit (%) | 1276 (65.5) | 612 (64.1) | 0.48^b^ |
| **Medical history** |  |  |  |
| history of hypertension (%) | 1662 (80.5) | 824 (79.8) | 0.69^b^ |
| history of atrial fibrillation (%) | 456 (22.6) | 230 (22.7) | 0.96^b^ |
| history of previous cerebrovascular event (%) | 405 (19.6) | 215 (20.8) | 0.46^b^ |
| history of diabetes mellitus (%) | 413 (20.0) | 204 (19.8) | 0.91^b^ |

**Table S1.** Comparison of baseline characteristics, initiated therapies, and medical history between the daytime (i.e. 7:00 AM to 10:59 PM) and nighttime (i.e. 11:00 PM to 06:59 AM) onset groups after propensity score matching. Because we anticipated more patients in the daytime group (16 hours) compared to the nighttime group (8 hours), we matched patients using a 2:1 ratio. Continuous variables without a normal distribution are presented as median (interquartile range). Categorical variables are expressed as number (n) with percentage (%) in parentheses. The p-values <0.05 that are considered statistically significant are depicted in a bold font. Abbreviations: GCS=Glasgow Coma Scale. ^a^Wilcoxon-Mann-Whitney-test, ^b^χ²-test.

|  | Nighttime  (n=1,032) | Daytime  (n=2,064) | p-Value |
| --- | --- | --- | --- |
| **Complications** |  |  |  |
| mortality (%) | (33.3) | (33.4) | 0.23 |
| cerebral oedema (%) | (8.7) | (8.0) | 0.10 |
| bleeding progression or rebleeding including secondary intraventricular hemorrhage (%) | (3.8) | (5.0) | 0.97 |
| pneumonia (%) | (20.1) | (18.5) | 0.45 |
| epileptic seizures (%) | (2.3) | (3.4) | 0.12 |
| hydrocephalus (%) | (4.4) | (5.0) | 0.72 |

**Table S2: Outcomes according to the time of day of ICH onset after propensity score matching.** The nighttime group summarizes patients suffering from ICH in the evening or night (11:00 PM- 6:59 AM); the daytime group summarises patients with ICH onset during the morning or midday (7:00 AM- 10:59 PM).
